# High titers of both rheumatoid factor and anti-CCP antibodies at baseline in patients with rheumatoid arthritis are associated with increased circulating baseline TNF level, low drug levels, and reduced clinical responses: a post hoc analysis of the RISING study

**DOI:** 10.1186/s13075-017-1401-2

**Published:** 2017-09-02

**Authors:** Tsutomu Takeuchi, Nobuyuki Miyasaka, Takashi Inui, Toshiro Yano, Toru Yoshinari, Tohru Abe, Takao Koike

**Affiliations:** 1 Division of Rheumatology, Department of Internal Medicine, School of Medicine, Keio University, 35 Shinanomachi, Shinjuku-ku, Tokyo, 160-8582 Japan; 2 Graduate School of Medical and Dental Sciences, Tokyo Medical and Dental University, 1-5-45, Yushima, Bunkyo-ku, Tokyo, 113-8519 Japan; 3 Ikuyaku Integrated Value Development Division, Mitsubishi Tanabe Pharma Corporation, 3-2-10, Dosho-machi, Chuo-ku, Osaka, 541-8505 Japan; 4 Saitama Medical Center, Saitama Medical University, 1981 Kamoda, Kawagoe, Saitama, 350-8550 Japan; 5 Sapporo Medical Center NTT EC, South-1 West-15, Chuo-ku, Sapporo, 060-0061 Japan

**Keywords:** Rheumatoid arthritis, Infliximab, Anticyclic citrullinated peptide antibodies, Rheumatoid factor, Clinical response, Pharmacokinetics, Prediction

## Abstract

**Background:**

Although both rheumatoid factor (RF) and anticyclic citrullinated peptide antibodies (anti-CCP) are useful for diagnosing rheumatoid arthritis (RA), the impact of these autoantibodies on the efficacy of tumor necrosis factor (TNF) inhibitors has been controversial. The aim of this post hoc analysis of a randomized double-blind study (the RISING study) was to investigate the influences of RF and anti-CCP on the clinical response to infliximab in patients with RA.

**Methods:**

Methotrexate-refractory patients with RA received 3 mg/kg of infliximab from weeks 0 to 6 and then 3, 6, or 10 mg/kg every 8 weeks from weeks 14 to 46. In this post hoc analysis, patients were stratified into three classes on the basis of baseline RF/anti-CCP titers: “low/low-C” (RF < 55 IU/ml, anti-CCP < 42 U/ml), “high/high-C” (RF ≥ 160 IU/ml, anti-CCP ≥ 100 U/ml), and “middle-C” (neither low/low-C nor high/high-C). Baseline plasma TNF level, serum infliximab level, and disease activity were compared between the three classes.

**Results:**

Baseline RF and anti-CCP titers showed significant correlations with baseline TNF and infliximab levels in weeks 2–14. Comparison of the three classes showed that baseline TNF level was lowest in the low/low-C group and highest in the high/high-C group (median 0.73 versus 1.15 pg/ml), that infliximab levels at week 14 were highest in the low/low-C group and lowest in the high/high-C group (median 1.0 versus 0.1 μg/ml), and that Disease Activity Score in 28 joints based on C-reactive protein at week 14 was lowest in the low/low-C group and highest in the high/high-C group (median 3.17 versus 3.82). A similar correlation was observed at week 54 in the 3 mg/kg dosing group, but not in the 6 or 10 mg/kg group. Significant decreases in both RF and anti-CCP were observed during infliximab treatment.

**Conclusions:**

RF/anti-CCP titers correlated with TNF level. This might explain the association of RF/anti-CCP with infliximab level and clinical response in patients with RA. Baseline RF/anti-CCP titers may serve as indices that aid infliximab treatment.

**Trial registration:**

ClinicalTrials.gov, NCT00691028. Retrospectively registered on 3 June 2008.

**Electronic supplementary material:**

The online version of this article (doi:10.1186/s13075-017-1401-2) contains supplementary material, which is available to authorized users.

## Background

Both rheumatoid factor (RF) and anticyclic citrullinated peptide antibodies (anti-CCP) are useful for diagnosing rheumatoid arthritis (RA) [1, 2], and they have been shown to be associated with the progression of joint destruction in patients with RA [3, 4]. However, the influence of these autoantibodies on the clinical status and disease activity of patients with RA has yet to be fully clarified.

RF and anti-CCP have recently been shown to influence the efficacy of some non-tumor necrosis factor (non-TNF) biological disease-modifying antirheumatic drugs (DMARDs) [5–9]. However, the influence of these autoantibodies on the efficacy of TNF inhibitors, the first biological DMARDs approved for RA, has been controversial [10–16].

The RISING study was a randomized, double-blind trial that demonstrated the usefulness of dose escalation of infliximab (IFX) in RA [17]. In a post hoc analysis of this study, we previously reported that the baseline plasma TNF level greatly influenced serum IFX levels and predicted clinical response at 1 year [18].

RF/anti-CCP double-positive but not single-positive patients with RA were reported to show significantly higher circulating TNF levels than double-negative patients [19]. We therefore hypothesized that “both RF-high and anti-CCP-high” would correlate with a higher TNF level, which would result in a lower IFX level and clinical response. In the present analysis using data from the RISING study, we explored the association between baseline RF and anti-CCP titers with efficacy of IFX therapy in patients with active RA despite methotrexate (MTX) treatment.

## Methods

### Study protocol

The protocol of the RISING study has been published elsewhere [17, 18]. Patients with active RA (diagnosed according to 1987 American College of Rheumatology criteria [20]) despite MTX treatment were treated with a standard dose (3 mg/kg) of an IFX originator (Remicade; Mitsubishi Tanabe Pharma Corporation, Osaka, Japan) at weeks 0, 2, and 6 (W0, W2, and W6, respectively), after which they were randomized to three dosing groups and treated with 3, 6, or 10 mg/kg of IFX every 8 weeks from W14 to W46. Active RA was defined by the presence of at least six swollen joints, at least six tender joints, and an erythrocyte sedimentation rate ≥ 28 mm/h or a serum C-reactive protein (CRP) level ≥ 2.0 mg/dl.

### Disease activity and laboratory testing

Disease activity was evaluated by the Disease Activity Score in 28 joints based on C-reactive protein (DAS28-CRP). Because there is controversy about the cutoff level of DAS28-CRP, which might underestimate disease activity when using cutoff values validated for DAS28 based on erythrocyte sedimentation rate [21], we used the cutoff levels that were previously estimated in Japanese patients with RA as follows: clinical remission (REM) < 2.3, low disease activity without clinical remission (LDA), ≥ 2.3 but < 2.7, moderate disease activity (MDA) ≥ 2.7 but ≤ 4.1, and high disease activity (HDA) > 4.1 [22].

RF titers were measured by a latex agglutination test using the Auto LIA-RF kit (Nissui Pharmaceutical Co., Tokyo, Japan) with a detectable limit of ≥ 3 IU/ml (normal range ≤ 15 IU/ml). Anti-CCP titers were measured by performing an enzyme-linked immunosorbent assay (ELISA) using the DIASTAT anti-CCP kit (Euro Diagnostica, Malmö, Sweden) with a detectable range of ≥ 0.6 to < 100 U/ml (normal range ≤ 5.0 U/ml). Disease activity, RF, and anti-CCP at W54 were evaluated using the last observation carried forward approach.

Baseline plasma TNF levels (just before the first IFX infusion) were measured by ELISA using the QuantiGlo ELISA Kit (QTA00B; R&D Systems Inc., Minneapolis, MN, USA) as described previously [18]. Baseline plasma interleukin (IL)-6 and serum matrix metalloproteinase (MMP)-3 levels were measured by ELISA [18]. All laboratory tests were performed at LSI Medience Corporation (Tokyo, Japan).

Serum IFX levels and anti-infliximab antibody (ATI) positivity were measured by ELISA [17] at Mitsubishi Tanabe Pharma Corporation using the same ELISA system (Janssen Biotech, Inc.; Horsham, PA, USA) as that used in previous phases II and III studies for RA [23–25]. IFX levels were evaluated at W2, W6, W10, W14, and W54, with the lower detection level of < 0.1 μg/ml. ATI positivity was evaluated at W54 in study completers or at 12 weeks after the last infusion in noncompleters. Patients with detectable serum IFX levels were considered to be ATI-negative and were not evaluated for ATI positivity (i.e., serum IFX levels were < 0.1 μg/ml in all ATI-positive patients), as described previously [17, 23–25].

### Stratification of patients using baseline RF and anti-CCP titers

Baseline RF and anti-CCP cutoff values for patient stratification were defined as follows: RF-low < 55 IU/ml and RF-high ≥ 160 IU/ml (both are tertile values at baseline in this study) and anti-CCP-low < 42 U/ml and anti-CCP-high ≥ 100 U/ml. The lower cutoff for anti-CCP of 42 U/ml was the first tertile for baseline anti-CCP titer, whereas the upper cutoff of 100 U/ml corresponded to the upper detection limit for anti-CCP (Additional file 1).

Patients with RA were then stratified into the following three classes on the basis of the above-mentioned cutoff values: low/low class (both RF-low and anti-CCP-low, low/low-C), high/high class (both RF-high and anti-CCP-high, high/high-C), and middle class (patients who did not meet the criteria for either class, middle-C) (Additional file 2). The following data were compared between the three classes: baseline plasma TNF level, serum IFX levels at W2, W6, W10, W14, and W54, and disease activities at W0, W2, W6, W10, W14, and W54.

### Statistical analysis

Spearman’s rank correlation test was used to evaluate the correlation of baseline RF titer and baseline anti-CCP titer with patient characteristics, serum IFX levels, and disease activity. The Kruskal-Wallis test or chi-square test was used to compare patients’ characteristics, IFX levels, and disease activity among the three dosing groups (3, 6, and 10 mg/kg) or among three classes stratified on the basis of RF/anti-CCP at baseline (low/low-C, middle-C, and high/high-C). In addition, RF titers, anti-CCP titers, and the rate of three classes at W30 and W54 were compared with those at baseline (W0) using the Wilcoxon signed-rank test in each dosing group. All statistical analyses were performed using SAS version 9.4 software (SAS Institute Japan Ltd., Tokyo, Japan). *p* < 0.05 (two-tailed) was considered to indicate statistical significance.

## Results

### Patient baseline characteristics and clinical response at week 54

Table 1 shows patient baseline characteristics and clinical response at W54 for each IFX dosing group. Median (IQR), first tertile, and second tertile of baseline RF titers were 92 (37–237), 55, and 160 IU/ml, respectively (Additional file 1). The proportion of patients who were RF-negative (≤15 IU/ml) was 13% (41 of 307). Although no significant difference was observed in patient baseline characteristics among three dosing groups, the RF-negative rate tended to be high in the 3 mg/kg group.

**Table 1:** Characteristics of patients at baseline and clinical response at week 54 in each dosing group

	3 mg/kg	6 mg/kg	10 mg/kg	*p* Value
At baseline (week 0)	(*n* = 99)	(*n* = 104)	(*n* = 104)	
Age, years	49.7 (11.7)	48.8 (11.8)	50.4 (12.5)	0.5370
Female sex	78 (79%)	86 (83%)	89 (86%)	0.4446^a^
Disease duration, years	8.3 (7.8)	7.2 (7.1)	8.4 (7.7)	0.4114
MTX dose, mg/week	7.8 (1.6)	7.9 (1.9)	7.7 (1.7)	0.6510
DAS28-CRP	5.59 (4.89, 6.31)	5.38 (4.85, 6.36)	5.50 (5.10, 6.11)	0.8026
TNF, pg/ml	0.92 (< 0.55, 1.29)	0.97 (0.70, 1.31)	0.89 (< 0.55, 1.24)	0.1320
RF				
Median (IQR), IU/ml	128 (27, 280)	82 (37, 273)	89 (43, 187)	0.6361
Range (minimum, maximum), IU/ml	< 3, 1700	< 3, 2340	< 3, 1950	
Negative (≤ 15 IU/ml)	19 (19%)	14 (13%)	8 (8%)	0.0551^a^
Anti-CCP				
Median (interquartile range), U/ml	≥ 100 (26, ≥ 100)	≥ 100 (23, ≥ 100)	≥ 100 (37, ≥ 100)	0.1308
Range (minimum, maximum), U/ml	< 0.6, ≥ 100	< 0.6, ≥ 100	< 0.6, ≥ 100	
Negative (≤ 5.0 U/ml)	9 (9%)	12 (12%)	4 (4%)	0.1171^a^
At week 54	(*n* = 99)	(*n* = 104)	(*n* = 104)	
DAS28-CRP at week 54	3.02 (2.13, 4.25)	2.72 (1.59, 3.99)	2.52 (1.69, 3.65)	0.0394
REM/LDA/MDA/HDA	30 (30%)/11 (11%)/27 (27%)/31 (31%)	41 (39%)/10 (10%)/29 (28%)/24 (23%)	47 (45%)/9 (9%)/31 (30%)/17 (16%)	0.0384
RF, IU/ml	61 (6, 152)	38 (8, 91)	42 (9, 76)	0.3386
Anti-CCP, U/ml	≥ 100 (12, ≥ 100)	47 (12, ≥ 100)	≥ 100 (24, ≥ 100)	0.1079

*Abbreviations: CCP*, Cyclic citrullinated peptide antibodies, *DAS28-CRP* Disease Activity Score in 28 joints based on C-reactive protein, *HDA* high disease activity, *LDA* Low disease activity without clinical remission, *MDA* Moderate disease activity, *MTX* Methotrexate, *REM* Clinical remission, *RF* Rheumatoid factor, *TNF* Tumor necrosis factor

Data are mean (SD), median (interquartile range), or number (%), unless otherwise described. The Kruskal-Wallis test was used to evaluate the differences among three dosing groups, except where indicated otherwise. Disease activity, RF, and anti-CCP at Week 54 were evaluated using the last observation carried forward approach. Cutoff values for DAS28-CRP were as follows: REM, <2.3; LDA, ≥2.3– < 2.7; MDA, ≥2.7– ≤ 4.1; HDA, > 4.1 [22]

^a^Chi-square test was used to evaluate the differences among three dosing-groups

The median (IQR) and first tertile of baseline anti-CCP titers were ≥ 100 (28, ≥ 100) and 42 U/ml, respectively. The proportion of patients with a baseline anti-CCP titer above the upper detection limit (≥ 100 U/ml) was 58% (177 of 307), and the proportion of patients who were anti-CCP-negative (≤ 5.0 U/ml) was 8% (25 of 307). The proportion of patients who were seronegative for both RF and anti-CCP was 6% (19 of 307). Comorbidity was observed in 78% of patients; the major comorbidities were hypertension (21%), pollinosis (17%), osteoporosis (13%), and anemia (12%).

Table 1 also shows the clinical responses of the 3, 6, and 10 mg/kg dosing groups at W54. Significant differences in DAS28-CRP and disease activity criteria at W54 were observed among the three dosing groups. In contrast, both RF and anti-CCP titers significantly decreased after IFX treatment in each dosing group; however, no significant difference was observed among the three dosing groups (Additional file 3).

### Correlations of baseline RF and anti-CCP titers with patient baseline characteristics

Table 2 shows the correlations of baseline RF and anti-CCP titers with patient baseline characteristics. The baseline RF titer showed significant correlations with sex, age, duration of disease, total modified Sharp score, MMP-3, and anti-CCP, as well as TNF level, although the correlation coefficient for each was low. In contrast, the baseline anti-CCP titer showed significant correlations with comorbidity and RF as well as TNF level. Accordingly, TNF level was the only baseline characteristic that correlated with both RF and anti-CCP.

**Table 2:** Correlation of rheumatoid factor and anti-cyclic citrullinated peptide antibodies with patient characteristics at baseline (week 0)

	RF at week 0		Anti-CCP at week 0	
	Rho	*p* Value	Rho	*p* Value
Sex (0 = male, 1 = female)	−0.157	0.0057	−0.043	0.4489
Age	0.154	0.0070	0.106	0.0643
BMI	0.037	0.5141	0.016	0.7863
Disease duration	0.119	0.0368	−0.046	0.4225
NSAID use^a^	−0.006	0.9119	0.022	0.7021
Glucocorticoid use^a^	0.022	0.6967	0.023	0.6893
DMARD (other than MTX) use^a^	−0.051	0.3727	0.065	0.2549
Duration of MTX use	0.050	0.3832	0.009	0.8687
MTX dose	−0.069	0.2256	−0.013	0.8184
Comorbidity^a^	−0.023	0.6874	0.129	0.0240
DAS28-CRP	0.087	0.1278	0.042	0.4596
Total modified Sharp score	0.132	0.0214	−0.019	0.7385
HAQ	0.089	0.1186	0.081	0.1557
MMP-3	−0.129	0.0236	−0.057	0.3198
IL-6	0.069	0.2287	0.040	0.4809
TNF	0.209	0.0002	0.117	0.0413
RF	–	–	0.373	<0.0001
Anti-CCP	0.373	<0.0001	–	–

*Abbreviations: BMI* Body mass index, *CCP* Cyclic citrullinated peptide antibodies, *DAS28-CRP* Disease Activity Score in 28 joints based on C-reactive protein, *HAQ* Health Assessment Questionnaire, *IL-6* Interleukin-6, *MMP-3* Matrix metalloproteinase-3, *MTX* Methotrexate, NSAID Nonsteroidal anti-inflammatory drug, *RF* Rheumatoid factor, *Rho* Spearman’s rank correlation coefficient, *TNF* Tumor necrosis factor

^a^Categories of response are 0 = no, 1 = yes

### Correlations of baseline RF and anti-CCP titers with serum IFX levels

We previously reported a significant negative correlation between the TNF level and IFX level [18]. In the present analysis, we explored the association of baseline RF and anti-CCP titers with IFX levels in W2 to W14 in patients receiving 3 mg/kg of IFX (Table 3). Similarly to our previous findings regarding TNF and IFX levels, significant negative correlations were noted between IFX levels and both baseline RF and anti-CCP titers at all time points (W2 to W14). Among the other patient baseline characteristics analyzed, only sex was significantly correlated with IFX levels at all time points.

**Table 3:** Correlation of rheumatoid factor and anti-cyclic citrullinated peptide antibodies at baseline with serum infliximab levels

	Serum infliximab level							
	Week 2		Week 6		Week 10		Week 14	
	Rho	*p* Value	Rho	*p* Value	Rho	*p* Value	Rho	*p* Value
Sex (0 = male, 1 = female)	0.302	< 0.0001	0.211	0.0002	0.190	0.0008	0.160	0.0049
Age	0.004	0.9381	0.064	0.2664	0.030	0.5965	0.004	0.9472
BMI	0.160	0.0049	0.054	0.3468	0.033	0.5620	−0.015	0.7946
Disease duration	0.098	0.0852	0.112	0.0490	0.106	0.0631	0.093	0.1022
NSAID use^a^	−0.087	0.1303	−0.018	0.7588	−0.039	0.4941	−0.060	0.2964
Glucocorticoid use^a^	−0.060	0.2908	−0.076	0.1822	−0.039	0.4920	−0.064	0.2635
DMARD (other than MTX) use^a^	−0.027	0.6361	−0.022	0.7002	−0.007	0.9018	0.027	0.6386
Duration of MTX use	0.100	0.0809	0.109	0.0558	0.063	0.2690	0.071	0.2141
MTX dose	−0.043	0.4554	−0.074	0.1946	−0.053	0.3550	−0.041	0.4730
Comorbidity^a^	0.063	0.2680	0.064	0.2626	0.118	0.0384	0.099	0.0823
DAS28-CRP	−0.128	0.0246	−0.116	0.0430	−0.125	0.0290	−0.073	0.2002
Total modified Sharp score	−0.049	0.3945	0.069	0.2271	0.059	0.3064	0.060	0.2979
HAQ	−0.104	0.0699	−0.035	0.5398	−0.060	0.2994	−0.048	0.4027
MMP-3	−0.107	0.0608	−0.146	0.0103	−0.103	0.0726	−0.101	0.0783
IL-6	−0.114	0.0461	−0.085	0.1357	−0.025	0.6639	0.000	0.9948
TNF	−0.150	0.0083	−0.163	0.0041	−0.169	0.0031	−0.158	0.0054
RF	−0.192	0.0007	−0.188	0.0009	−0.201	0.0004	−0.222	< 0.0001
Anti-CCP	−0.153	0.0071	−0.121	0.0344	−0.133	0.0195	−0.172	0.0025

*Abbreviations: BMI* Body mass index, *CCP* Cyclic citrullinated peptide antibodies, *DAS28-CRP* Disease Activity Score in 28 joints based on C-reactive protein, *HAQ* Health Assessment Questionnaire, *IL-6* Interleukin-6, *MMP-3* Matrix metalloproteinase-3, *MTX* Methotrexate, NSAID Nonsteroidal anti-inflammatory drug, *RF* Rheumatoid factor, *Rho* Spearman’s rank correlation coefficient, *TNF* Tumor necrosis factor

^a^Categories of response are 0 = no, 1 = yes

### Correlation of patient baseline characteristics with the three classes stratified by baseline RF and anti-CCP titers

We initially hypothesized that TNF level should be low in patients who are negative for RF and anti-CCP at baseline, which would lead to high IFX levels. However, the relatively small patient group of 41 RF-negative patients and 25 anti-CCP-negative patients in the RISING study prevented analysis of differences in IFX level and disease activity at W54 between the three IFX dosing groups. To resolve this issue, we stratified the patients in the RISING study into three classes using cutoff values for both RF and anti-CCP as described in the Methods section above as low/low-C (RF-low/anti-CCP-low), high/high-C (RF-high/anti-CCP-high), and middle-C (those who did not meet the criteria for either class) (Additional file 2).

Table 4 shows patient baseline characteristics in the three stratified classes. A significant difference was observed in baseline TNF levels among three classes, with the TNF level being lowest in low/low-C (median 0.73 pg/ml), middle in middle-C (median 0.91 pg/ml), and highest in high/high-C (median 1.15 pg/ml). The proportions of patients with a high baseline TNF level ≥ 1.65 pg/ml [18] in low/low-C, middle-C, and high/high-C were 8%, 8%, and 30%, respectively. Regarding disease activity and Health Assessment Questionnaire at baseline, significant differences were observed among the three classes; however, the values were lower in middle-C.

**Table 4:** Patient characteristics of the three classes stratified by rheumatoid factor and anti-cyclic citrullinated peptide antibodies

	Low/low class (*n* = 53)	Middle class (*n* = 183)	High/high class (*n* = 71)	*p* Value
RF, IU/ml	11 (5, 30)	87 (50, 139)	384 (248, 604)	< 0.0001
Anti-CCP, U/ml	7 (2, 22)	≥ 100 (38, ≥ 100)	≥ 100 (≥ 100, ≥ 100)	< 0.0001
Female sex	89%	83%	76%	0.1766^a^
Age, years	46.4 (13.0)	49.3 (12.1)	53.0 (10.3)	0.0093
BMI	22.2 (4.2)	22.0 (3.3)	22.8 (3.9)	0.3774
Disease duration	7.3 (6.9)	7.9 (7.2)	8.6 (8.7)	0.8541
NSAID use	89%	87%	90%	0.8299^a^
Glucocorticoid use	66%	70%	65%	0.6263^a^
DMARD (other than MTX) use	32%	31%	31%	0.9793^a^
MTX dose, mg/week	7.6 (1.7)	7.9 (1.8)	7.7 (1.7)	0.5264
Duration of MTX use	2.7 (2.6)	2.7 (2.8)	2.6 (2.8)	0.9885
Comorbidity	77%	74%	87%	0.0810^a^
DAS28-CRP	5.54 (4.92, 6.42)	5.39 (4.89, 6.01)	5.74 (5.12, 6.37)	0.0251
Total modified Sharp score	36.0 (8.0, 56.8)	36.5 (12.0, 74.1)	31.5 (13.0, 57.0)	0.4305
HAQ	1.21 (0.68)	1.11 (0.63)	1.38 (0.67)	0.0132
MMP-3, ng/ml	262 (132, 561)	207 (105, 420)	196 (101, 352)	0.2372
IL-6, pg/ml	26.5 (12.3, 56.5)	28.5 (12.4, 70.2)	33.6 (15.1, 61.8)	0.6163
TNF, pg/ml	0.73 (< 0.55, 1.09)	0.91 (< 0.55, 1.23)	1.15 (0.72, 1.82)	0.0002
Low/intermediate/high^b^	38%/55%/8%	29%/63%/8%	20%/51%/30%	0.0007

*Abbreviations: BMI* Body mass index, *CCP* Cyclic citrullinated peptide antibodies, *DAS28-CRP* Disease Activity Score in 28 joints based on C-reactive protein, *HAQ* Health Assessment Questionnaire, *IL-6* Interleukin-6, *MMP-3* Matrix metalloproteinase-3, *MTX* Methotrexate, NSAID Nonsteroidal anti-inflammatory drug, *RF* Rheumatoid factor, *Rho* Spearman’s rank correlation coefficient, *TNF* Tumor necrosis factor

Data are mean (SD), median (interquartile range), or patients rate (%). The Kruskal-Wallis test was used to evaluate the differences among three classes unless otherwise noted

^a^Chi-square test was used to evaluate the differences among three classes

^b^Distribution of TNF levels were defined as follows: low < 0.55, intermediate ≥ 0.55 to < 1.65, and high ≥ 1.65 pg/ml [18]

### Correlation of serum IFX levels and disease activity until week 14 in the three stratified classes

Table 5 shows the IFX levels in W2 to W14 and disease activity in W0 to W14 in the three classes. Significant differences in IFX levels were observed in W2 to W14, with observed levels highest in low/low-C and lowest in high/high-C. Even in high/high-C, median IFX levels in W2 to W10 (2 or 4 weeks after the previous infusion) were above the threshold level for clinical response (≥ 1.0 μg/ml), the value for which has been reported in previous clinical studies using the same ELISA system [17, 24, 25]. However, in high/high-C, the median IFX level at W14 was 0.4 μg/ml, and the proportion of patients with IFX levels ≥ 1.0 μg/ml was only 30%. The corresponding proportion in middle-C was between that in low/low-C and that in high/high-C for each time point.

**Table 5:** Serum infliximab level and disease activity in three classes stratified on the basis of rheumatoid factor and anti-cyclic citrullinated peptide antibodies

	Low/low class (*n* = 53)	Middle class (*n* = 183)	High/high class (*n* = 71)	*p* Value
Serum infliximab level, μg/ml				
Week 2	13.9 (12.0, 15.7)	13.2 (10.2, 16.3)	12.0 (9.5, 14.2)	0.0028
Week 6	7.6 (4.5, 10.8)	7.1 (2.5, 10.0)	5.0 (1.6, 8.2)	0.0072
Week 10	7.7 (3.9, 10.5)	6.3 (2.0, 10.3)	3.8 (1.5, 7.9)	0.0040
Week 14	1.5 (0.6, 2.1)	0.7 (<0.1, 2.0)	0.4 (<0.1, 1.1)	0.0014
Week 14, < 0.1/≥ 0.1 to < 0.1/≥ 1.0	13%/25%/62%	33%/25%/42%	32%/38%/30%	0.0015
DAS28-CRP				
Week 2	3.89 (3.09, 4.51)	3.90 (3.24, 4.52)	4.01 (3.34, 4.79)	0.2770
Week 6	3.32 (2.78, 4.16)	3.42 (2.67, 4.22)	3.68 (2.96, 4.36)	0.2381
Week 10	3.19 (2.57, 3.77)	3.24 (2.55, 4.12)	3.48 (2.67, 4.20)	0.3612
Week 14	3.17 (2.49, 3.69)	3.53 (2.63, 4.36)	3.82 (2.62, 4.97)	0.0764
Week 14, REM/LDA/MDA/HDA	17%/15%/51%/17%	15%/12%/40%/33%	18%/10%/31%/41%	0.1455

*Abbreviations: DAS28-CRP* Disease Activity Score in 28 joints based on C-reactive protein, *HDA* High disease activity, *LDA* Low disease activity without clinical remission, *MDA* Moderate disease activity, *REM* Clinical remission

Data are median (interquartile range) or number of patients (%). Cutoff values for DAS28-CRP were as follows: REM < 2.3, LDA ≥ 2.3 to < 2.7, MDA ≥ 2.7 to ≤ 4.1, HDA > 4.1 [22]. The Kruskal-Wallis test was used to evaluate the differences among three classes

With regard to disease activity, an opposite trend was observed: The median DAS28-CRP was lowest in low/low-C and highest in high/high-C, although the difference (marginally significant) was observed only at W14 (8 weeks after the previous infusion).

### Correlation of serum IFX levels and disease activity at week 54 with the three stratified classes in each IFX dosing group

**Fig. 1:**
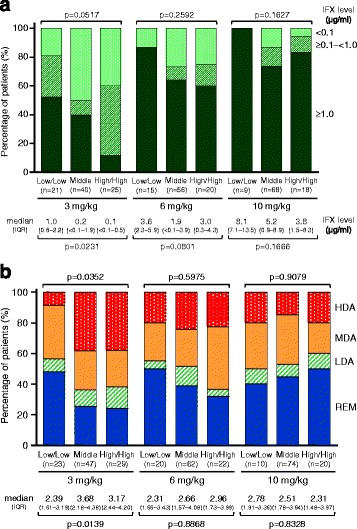
**a**, **b** Serum infliximab level and disease activity at week 54 in three stratified classes. Differences among three classes stratified on the basis of rheumatoid factor and anti-cyclic citrullinated peptide antibodies at week 0 in serum infliximab (IFX) level at week 54 (**a**) and with disease activity at week 54 **b** were evaluated by Kruskal-Wallis test. Disease activity was evaluated using Disease Activity Score in 28 joints based on C-reactive protein with the following REM cutoff levels: < 2.3, LDA ≥ 2.3 but < 2.7, MDA ≥ 2.7 but ≤ 4.1, and HDA > 4.1 [22]. *HDA* High disease activity, *LDA* Low disease activity without clinical remission, *MDA* Moderate disease activity, *REM* Clinical remission

Figure 1a and b shows the IFX levels and DAS28-CRP at W54 in the three stratified classes in each IFX dosing group (3, 6, or 10 mg/kg group). In the 3 mg/kg dosing group, a significant difference in IFX levels at W54 was observed among the three stratified classes. The proportion of patients with IFX level ≥ 1.0 μg/ml at W54 was 52% in low/low-C but only 12% in high/high-C with a median IFX level of 0.1 μg/ml (close to the lower detection limit).

A significant difference was also observed in disease activity at W54 in patients receiving 3 mg/kg of IFX. The proportions of patients with REM and HDA were 48% and 9% in low/low-C and 24% and 38% in high/high-C, respectively. In patients receiving IFX at 6 or 10 mg/kg, the IFX level at W54 was lowest in high/high-C, but not to a significant degree. The median IFX level in high/high-C at W54 was 3.0 or 3.8 μg/ml in patients receiving 6 or 10 mg/kg of IFX, respectively, either of which was ≥ 1.0 μg/ml. With regard to disease activity at W54 in patients receiving IFX at 6 or 10 mg/kg, no significant difference was observed among the three classes. In addition, the proportion of patients with RF-high/anti-CCP-high decreased, and the proportion of those with RF-low/anti-CCP-low increased during IFX treatment in each dosing group, despite no differences among the three dosing groups (Additional file 3).

### ATI positivity

As described in the Methods section above, patients with detectable serum IFX levels (≥ 0.1 μg/ml) were defined as ATI-negative and were not evaluated for ATI positivity. ATI positivity was evaluated in 98 patients. ATI positivity rates in low/low-C, middle-C, and high/high-C were 13% (3 of 23, ATI was analyzed in 5), 34% (16 of 47, ATI was analyzed in 26), and 28% (8 of 29, ATI was analyzed in 14), respectively, in the 3 mg/kg dosing group (*n* = 99); 15% (3 of 20, ATI was analyzed in 6), 24% (15 of 62, ATI was analyzed in 21), and 27% (6 of 22, ATI was analyzed in 7), respectively, in the 6 mg/kg group (*n* = 104); and 10% (1 of 10, ATI was analyzed in 1), 14% (10 of 74, ATI was analyzed in 14), and 10% (2 of 20, ATI was analyzed in 4), respectively, in the 10 mg/kg group (*n* = 104). ATI positivity rates in low/low-C tended to be lower than in the other classes, but no significant difference was observed among the three dosing groups.

## Discussion

Despite its efficacy in treating RA [26–29], IFX treatment is very costly. Therefore, predicting the clinical efficacy of IFX therapy is extremely important with regard to medical economics. Although prediction of the clinical efficacy of IFX therapy has been investigated in many studies, results have been controversial [10, 11, 30]. Although researchers in some studies reported that early response predicted long-term responses [31, 32], identifying predictive factors “at baseline” would be even more worthwhile in establishing IFX treatment strategies.

Our previous findings derived from a post hoc analysis of the RISING study demonstrated that patients with a high baseline TNF level had low IFX levels during IFX therapy and that this was associated with a poor clinical response 1 year later, particularly in patients on 3 mg/kg therapy [18]. However, TNF levels are generally quite low, and measurement results can differ markedly depending on the assay system used [18, 33–35]. In addition, measurement of TNF levels is not routine in clinical practice. Thus, other factors that can easily be measured in the clinical setting and can predict the efficacy of IFX therapy are desired.

In this post hoc analysis of the RISING study, we found a positive correlation of baseline TNF level, and a negative correlation of IFX levels, in W2 to W14 with baseline RF and anti-CCP titers. We then stratified patients using baseline RF and anti-CCP titers into low/low-C, middle-C, and high/high-C to evaluate the correlation with TNF levels as well as IFX levels and clinical responses. We found that RF-high/anti-CCP-high patients (high/high-C) had higher baseline TNF and lower IFX levels than other classes (Tables 4 and 5).

In RF-high/anti-CCP-high patients, humoral immunity may be enhanced, leading to an increasing risk of ATI expression, which correlates with lower IFX levels [36, 37]. Indeed, ATI positivity was higher in high/high-C than in low/low-C in the 3 and 6 mg/kg dosing groups, despite the absence of a significant difference. Therefore, the low IFX level in high/high-C might be due to the induction of ATI production in this patient class. Meanwhile, we observed a negative correlation between baseline RF/anti-CCP and IFX level even at the early induction phase (W2, after the first infusion), in which most patients were thought not to produce ATI (Table 5). We therefore considered that the correlation of baseline RF/anti-CCP with IFX level is likely due to the high baseline TNF level and that high ATI positivity in high/high-C may be the consequence of a low IFX level. However, we did not evaluate ATI positivity in patients with a detectable serum IFX level, and we did not exclude the effect of ATI production on the low IFX level.

Upon evaluating the correlation between DAS28-CRP until W14 and the three stratified classes, a significant correlation was observed in only at W14 (8 weeks after the previous infusion) (Table 5). Although IFX levels at W2 to W10 were lower in high/high-C than in the other classes, ≥ 60% of patients in high/high-C had a serum level > 1.0 μg/ml, which was reported as the threshold value for clinical response in several clinical studies using the same ELISA system [17, 24, 25]. In contrast, the median serum IFX level at W14 was 0.4 μg/ml, and approximately one-third of patients showed a level that was below the lower limit of detection (< 0.1 μg/ml). Given these findings, we believe that the difference (marginally significant) in disease activity observed only at W14 was likely due to the markedly low IFX levels at W14 in high/high-C.

Similarly to these findings at W14, significant differences in IFX levels and disease activity were also observed at W54 in patients who continued to receive IFX at 3 mg/kg (Fig. 1a and b): high IFX levels and LDA in low/low-C, low IFX levels and HDA in high/high-C, and intermediate values in middle-C. Given these findings, baseline RF and anti-CCP titers were clearly associated with treatment response at W54 in patients receiving 3 mg/kg IFX.

In contrast, in patients receiving IFX at 6 or 10 mg/kg, IFX level at W54 tended to be lowest in high/high-C. However, no significant difference was observed in either IFX level or disease activity. In high/high-C, the median IFX levels at W54 in the 6 and 10 mg/kg dosing groups were 3.0 and 3.8 μg/ml, respectively, and the proportions of patients with a level above the threshold for clinical response (≥ 1.0 μg/ml) at W54 were 60% and 80%, respectively. Given these findings, the lack of difference in disease activity at W54 in the 6 and 10 mg/kg dosing groups was likely due to the sufficiently high IFX levels observed even in high/high-C.

Upon comparing disease activity criteria between the three IFX dosing groups (3, 6, and 10 mg/kg), in each of the three classes, no significant difference was observed in low/low-C (*p* = 0.845 by Kruskal-Wallis test), whereas some degree of difference was observed in high/high-C (*p =* 0.183) and middle-C (*p* = 0.026). These findings suggest that dose escalation of IFX might not be clinically meaningful in low/low-C, most of whom maintained the threshold for clinical response. Dose escalation would therefore be more effective in middle-C and high/high-C.

In addition to baseline RF and anti-CCP titers, sex was found to be significantly correlated with IFX levels in W2 to W14; indeed, IFX levels tended to be higher in female than in male patients. However, baseline RF titers were significantly lower in female patients, and baseline TNF levels tended to be lower in female than in male patients (median 0.90 versus 1.03 pg/ml). In addition, the female-to-male ratio in high/high-C was lower than in low/low-C (data not shown). Differences in baseline RF and TNF levels and in female-to-male ratio among the classes might have contributed to the apparent correlations between sex and IFX levels.

Although details regarding the mechanism underlying the correlation between RF/anti-CCP and TNF levels are unknown, a previous in vitro study showed that anti-CCP induced the production of inflammatory cytokines in the synovial membrane and that this was amplified by RF [38]. This finding suggests that the induction of TNF production in the synovial membrane may elevate circulating TNF levels in patients with high RF/anti-CCP titers. In this study, RF and anti-CCP titers at W54 were significantly decreased in all three IFX dosing groups compared with those at W0 (Additional file 3), suggesting that a “vicious cycle” might exist in patients with RA whereby RF/anti-CCP promotes TNF production, which in turn contributes to further induction of RF/anti-CCP through an as yet unknown mechanism.

In the BeSt Study, patients who sustained drug-free remission were reported to have significantly lower baseline RF and anti-CCP titers than those who did not [39]. In patients in whom RF and anti-CCP titers returned to the normal range with IFX therapy, the aforementioned “vicious cycle” might have been stopped. This stop might enable tapering of IFX in these patients with RA (i.e., dose reduction or withdrawal). However, further study is needed to prove this hypothesis.

Some IFX biosimilars, which are available for rheumatological as well as dermatological and gastroenterological conditions in some countries, have been reported to show the same efficacy, safety, and pharmacokinetics as the IFX originator Remicade [40]. Therefore, our results might theoretically be applicable to these IFX biosimilars. However, we used only the IFX originator in this study, and further study is needed to confirm the adaptation.

Several limitations of this study warrant mention. The first and most critical limitation is that the upper detection limit for anti-CCP was 100 U/ml, and baseline anti-CCP titers were ≥ 100 U/ml in 58% of patients in this study. Because of this low upper limit, we could not stratify three classes using a “true second tertile” of anti-CCP (58% were stratified as anti-CCP-high), and analyses in patients with an extremely high anti-CCP titer were difficult. Second, we could not delineate each influence of RF or anti-CCP on TNF level, IFX levels, or disease activity, because RF and anti-CCP were significantly correlated (rho = 0.373, *p* < 0.0001) and resulted in the small numbers of patients with RF-high/anti-CCP-low or RF-low/anti-CCP-high. Third, TNF level could not be explained solely using RF and anti-CCP titers. Despite the significant correlation of RF/anti-CCP titers with TNF level, the correlation coefficients were low. In addition, TNF level was also significantly correlated with other baseline characteristics, such as DAS28-CRP, age, and IL-6 level. Accordingly, we cannot rule out the possibility that other factors besides RF/anti-CCP titers influenced the TNF level. Fourth, IFX levels may not actually be linked to baseline RF/anti-CCP titers, but may be linked to RF/anti-CCP titers at the point of measurement. Although both RF and anti-CCP titers were significantly reduced in all three dosing groups after IFX treatment (Additional file 3), both autoantibody levels at baseline and W54 were closely correlated in this study (rho = 0.819 for RF, rho = 0.871 for anti-CCP). In addition, IFX levels at W54 tended to be more strongly correlated with RF and anti-CCP titers at W54 than at W0 (data not shown). Under these conditions, some inaccuracy may exist when predicting clinical response on the basis of baseline RF/anti-CCP titers in certain patient populations, such as those showing extreme or no change of RF/anti-CCP titers regardless of their clinical response during IFX therapy. Allowing for these limitations, these findings will provide useful indices for IFX treatment strategy. However, given that the RISING study did not measure circulating autoantibodies other than RF and anti-CCP, future studies may be required to clarify the influence of other autoantibodies on IFX levels and clinical responses in patients with RA.

## Conclusions

The findings of our present post hoc analysis of the RISING study demonstrated that baseline RF and anti-CCP titers are associated with both TNF level at baseline and IFX levels during subsequent IFX therapy, as well as that clinical responses are predictable to some degree before the start of IFX therapy. These findings will provide useful indices in implementing a strategy for long-term use of IFX in accordance with treat-to-target strategy [41, 42].

## Additional files

Additional file 1: Distribution of RF and anti-CCP titers at week 0 in all patients (*n* = 307). (PDF 39 kb)

Additional file 2: Stratification of patients based on RF and anti-CCP titers at week 0. (PDF 419 kb)

Additional file 3: RF and anti-CCP titers at weeks 0, 30, and 54. (PDF 479 kb)

## Abbreviations

ATI: Anti-infliximab antibody
BMI: Body mass index
CCP: Cyclic citrullinated peptide
CRP: C-reactive protein
DAS28-CRP: Disease Activity Score in 28 joints based on C-reactive protein
DMARD: Disease-modifying antirheumatic drug
ELISA: Enzyme-linked immunosorbent assay
HAQ: Health Assessment Questionnaire
HDA: High disease activity
IFX: Infliximab
IL: Interleukin
LDA: Low disease activity without clinical remission
MDA: Moderate disease activity
MMP: Matrix metalloproteinase
MTX: Methotrexate
NSAID: Nonsteroidal anti-inflammatory drug
RA: Rheumatoid arthritis
REM: Clinical remission
RF: Rheumatoid factor
TNF: Tumor necrosis factor
