# Supplementary material for: High titers of both rheumatoid factor and anti-CCP antibodies at baseline in patients with rheumatoid arthritis are associated with increased circulating baseline TNF level, low drug levels, and reduced clinical responses: a post hoc analysis of the RISING study
Source: Arthritis Res Ther. 2017 Sep 2;19:194. doi: 10.1186/s13075-017-1401-2 (PMC5581496; doi:10.1186/s13075-017-1401-2)
Supplement: Supplementary file 1 — Distribution of RF and anti-CCP titers at week 0 in all patients (n = 307). (PDF 39 kb) [file 13075_2017_1401_MOESM1_ESM.pdf]

Additional file 1. Distribution of RF and anti-CCP titers at Week 0 in all patients (n=307)

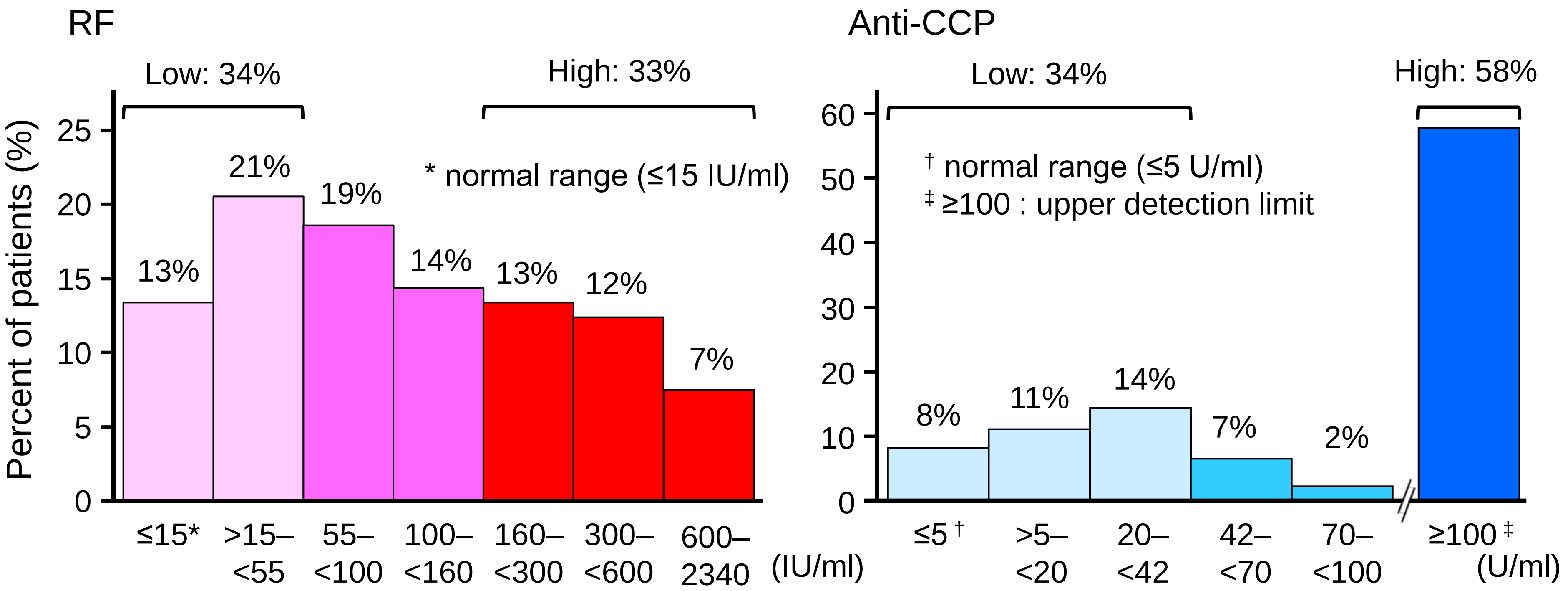

RF titers were measured by a latex agglutination test with a detectable limit of ≥3 IU/ml (normal range: ≤15 IU/ml). Anti-CCP titers were measured by an enzyme-linked immunosorbent assay (ELISA) with a detectable range of ≥0.6–<100 U/ml (normal range: ≤5.0 U/ml).
