# Supplementary material for: High titers of both rheumatoid factor and anti-CCP antibodies at baseline in patients with rheumatoid arthritis are associated with increased circulating baseline TNF level, low drug levels, and reduced clinical responses: a post hoc analysis of the RISING study
Source: Arthritis Res Ther. 2017 Sep 2;19:194. doi: 10.1186/s13075-017-1401-2 (PMC5581496; doi:10.1186/s13075-017-1401-2)
Supplement: Supplementary file 2 — Stratification of patients based on RF and anti-CCP titers at week 0. (PDF 419 kb) [file 13075_2017_1401_MOESM2_ESM.pdf]

Additional file 2. Stratification of patients based on RF and anti-CCP titers at Week 0

		RF at Week 0 (IU/ml)		
		<55 (n=104)	≥55–<160 (n=101)	≥160 (n=102)
Anti-CCP at Week 0 (U/ml)	<42 (n=103)	Low/Low-C (n=53)	Middle-C (n=183)	
	≥42–<100 (n=27)			
	≥100 (n=177)			High/High-C (n=71)

RA patients were stratified into the following 3 classes based on RF and anti-CCP titers at Week 0.

- Low/Low-C (both RF: <55 IU/ml, and anti-CCP: <42 U/ml)
- High/High-C (both RF: ≥160 IU/ml, and anti-CCP: ≥100 U/ml)
- Middle-C (patients who did not meet the criteria for either class)
