# Supplementary material for: High titers of both rheumatoid factor and anti-CCP antibodies at baseline in patients with rheumatoid arthritis are associated with increased circulating baseline TNF level, low drug levels, and reduced clinical responses: a post hoc analysis of the RISING study
Source: Arthritis Res Ther. 2017 Sep 2;19:194. doi: 10.1186/s13075-017-1401-2 (PMC5581496; doi:10.1186/s13075-017-1401-2)
Supplement: Supplementary file 3 — RF and anti-CCP titers at weeks 0, 30, and 54. (PDF 479 kb) [file 13075_2017_1401_MOESM3_ESM.pdf]

Additional file 3. RF and anti-CCP titers at Weeks 0, 30, and 54

	All (n=307)	3 mg/kg (n=99)	6 mg/kg (n=104)	10 mg/kg (n=104)	p
RF, IU/ml					
Week 0	92 (37, 237)	128 (27, 280)	82 (37, 273)	89 (43, 187)	0.6361
Week 30 [†‡]	47 (12, 99)	69 (8, 137)	37 (13, 96)	47 (12, 87)	0.3794
Week 54 [‡]	43 (8, 96)	61 (6, 152)	38 (8, 91)	42 (9, 76)	0.3386
Anti-CCP, U/ml					
Week 0	≥100 (28, ≥100)	≥100 (26, ≥100)	≥100 (23, ≥100)	≥100 (37, ≥100)	0.1308
Week 30 [†‡]	75 (16, ≥100)	≥100 (15, ≥100)	59 (15, ≥100)	91 (22, ≥100)	0.5064
Week 54 [‡]	64 (15, ≥100)	≥100 (12, ≥100)	47 (12, ≥100)	≥100 (24, ≥100)	0.1079
Rate of Low/Low-C, Middle-C, and High/High-C patients [§]					
Week 0	17% / 60% / 23%	23% / 47% / 29%	19% / 60% / 21%	10% / 71% / 19%	0.7069
Week 30 [†‡]	28% / 60% / 12%	30% / 51% / 19%	30% / 57% / 13%	23% / 71% / 5%	0.7555
Week 54 [‡]	32% / 57% / 11%	32% / 48% / 19%	36% / 54% / 11%	28% / 68% / 4%	0.4501

Data are median (interquartile range), or patients rate (%). RF, and anti-CCP at Week 54 were evaluated using the last observation carried forward approach. Kruskal-Wallis test was used to investigate the difference among 3 dosing-groups; [†] n=287; [§] Low/Low-C, RF: <55 IU/ml and anti-CCP: <42 U/ml; Middle-C, neither Low/Low-C nor High/High-C; High/High-C, RF: ≥160 IU/ml and anti-CCP: ≥100 U/ml; in each time-point. [‡] Significant differences were observed in RF, anti-CCP and rates of Low/Low-C, Middle-C, and High/High-C versus those at baseline (Week 0) in every dosing-group (p<0.01, Wilcoxon singed-rank test).
